# The impact of treatment modality on quality of life in glaucoma patients: findings from a clinical survey study in Augusta, GA

**DOI:** 10.1007/s10792-025-03895-7

**Published:** 2025-12-29

**Authors:** Neel Edupuganti, Haley Chishom, Tommy Bui, Danny Alevy, Tae Jin Lee, Mathilde Franklin, Marc Töteberg-Harms

**Affiliations:** 1 Medical College of Georgia, Department of Ophthalmology, Augusta University, Augusta, GA USA; 2 Medical College of Georgia, Center for Biotechnology and Genomic Medicine, Augusta University, Augusta, GA USA; 3 Department of Ophthalmology, Casey Eye Institute, Oregon and Health Science University, Portland, OR USA; 4 Eastern Virginia medical school, Department of Ophthalmology, Norfolk, VA USA; 5 University of Iowa, Carver College of Medicine, Department of Ophthalmology & Visual Sciences, Iowa City, IA USA

**Keywords:** Glaucoma treatment, Quality-of-life, Visual function, Intraocular pressure

## Abstract

**Purpose:**

This study evaluates the impact of medical versus combined medical and surgical glaucoma treatments on patients’ quality of life (QoL). While both approaches reduce intraocular pressure, research shows mixed effects on QoL. Given updated clinical guidelines and the gap in QoL-focused glaucoma management, this study applies validated QoL measures to assess treatment effects and inform patient-centered care.

**Methods:**

From June to October 2023, 180 patients at Augusta University Eye Care Center completed the 25-item National Eye Institute Visual Function Questionnaire (NEI VFQ-25) and Glaucoma Profile Instrument (GPI) surveys. Chart reviews provided demographic and clinical data, including glaucoma status and treatment. QoL scores were calculated using validated algorithms. Associations between clinical variables and QoL outcomes were analyzed using ANOVA and t-tests in R 4.4.1.

**Results:**

Among 180 patients, 103 had glaucoma or were suspects. 64% of glaucoma patients received medical treatment only, and 34% received combined medical and surgical treatment. VFQ-25 scores differed significantly by race (*p* = 0.0489), severity (*p* = 0.0001), and age (*p* = 0.0459), but not by gender, glaucoma status, or treatment. Severe glaucoma patients had the lowest QoL. Severity strongly impacted GPI scores (*p* = 0.0092) and multiple VFQ-25 domains. Differences by treatment modality were observed only in the driving subscale (*p* = 0.0378). No significant differences were observed in overall QoL scores between treatment groups.

**Conclusion:**

No significant vision-related QoL differences were found between treatment groups. Most patients were medically managed, possibly reflecting limited QoL benefit from surgery. QoL correlated more with disease severity than treatment modality or demographics. These findings highlight a disconnect between current treatment strategies and patient-perceived outcomes, underscoring the need for innovative strategies to improve QoL in glaucoma care.

**Supplementary Information:**

The online version contains supplementary material available at 10.1007/s10792-025-03895-7.

## Introduction

Glaucoma is the leading cause of irreversible blindness worldwide, characterized by loss of retinal ganglion cells and cupping of the optic disc. While elevated intraocular pressure (IOP) is a major risk factor for glaucoma, some patients may develop glaucoma without elevated IOP [1]. Primary open-angle glaucoma (POAG) is the most prevalent form of glaucoma in the United States [1]. POAG can occur insidiously, with most patients being asymptomatic in the early stages of the disease [2]. As the optic nerve damage progresses, peripheral visual fields are diminished initially, with further progression resulting in central vision loss. This pattern of peripheral to central vision may not be noticed by patients until they reach the severe stages of disease [1].

Given the significant impact of glaucoma on daily functioning and mental health, preserving quality of life (QoL), in addition to maintaining visual function, is a primary goal of treatment and management [2]. Patients with glaucoma often report reduced vision-related QoL compared to the general population, particularly in patients with limited visual acuity and visual field loss [3–6]. Gradual vision deterioration leads to reduced QoL due to loss of independence in performing activities of daily living, such as driving, shopping, and reading [7]. Many studies have found that a patient’s QoL and glaucoma severity are inversely correlated [6–8]. They also have indicated that poor quality of health may be associated with patients’ perception of their illness severity, as patients with more positive views of their illness experience greater QoL [3, 9].

Treatment options for glaucoma vary by disease severity, and these options include medical, surgical, or combined approaches [10]. Studies comparing surgical versus medical management have shown that patients who undergo trabeculectomy tend to have lower mean intraocular pressures following surgery, and thus, slower visual field loss as compared to medical treatment [11]. However, there is no clear association in the change in QoL [11, 12]. One study in Nigeria reported lower QoL among surgically treated patients [13], while the Collaborative Initial Glaucoma Treatment Study (CIGTS) found no significant difference at 5 years post-operatively [14].

Both medical and surgical treatments can affect patient independence and vision-related QoL, with medication burden and surgical risks such as cataract formation contributing negatively [11, 12, 15, 16]. Some studies suggest surgery may negatively impact QoL in early glaucoma, but QoL scores are similar between both treatment types in moderate to advanced stages [17]. Interestingly, only surgical treatment showed higher National Eye Institute Visual Function Questionnaire (NEI VFQ) general health scores compared to combined medical and surgical treatment [15]. Furthermore, demographic factors influence outcomes. Older adults reported less functional disability, and women were more likely to report greater symptoms than men from surgical treatment [18].

Traditionally, glaucoma treatment success has been measured via IOP reduction and visual field preservation [19–25]. Many landmark trials such as the Collaborative Normal Tension Glaucoma Study (CNTGS), the Ocular Hypertension Study (OHTS), and the European Glaucoma Prevention Study (EGPS) showed that there are many benefits of lowering IOP and guided treatment based on disease severity and patient characteristics [19–21]. The Early Manifest Glaucoma Trial (EMGT) and the United Kingdom Glaucoma Treatment Study (UKGTS) found that early treatment of open-angle glaucoma delays glaucoma progression [22, 23]. In patients with more advanced glaucoma, the Advanced Glaucoma Intervention Study (AGIS) and the CIGTS compared medical and surgical management, helping guide treatment selection based on severity and patient profile [24, 25].

Despite these advancements in glaucoma management, there remains a significant gap in understanding and consistently evaluating the impact of treatment on patients’ QoL. According to the European Glaucoma Society’s 2021 guidelines, the primary goal of glaucoma management has advanced to include maintaining patients’ quality of life along with preserving visual function and minimizing treatment burden [26]. The current state of glaucoma management is to follow clinical and diagnostic factors for glaucoma progression, but there is no established methodology to follow QoL as a foundation for treatment management. Currently, visual field status serves as the most approximate clinical indicator of QoL; however, there is unclear utility and guidance for treatment based on following QoL in patients with glaucoma [4]. Therefore, we aim to help bridge this lack of understanding by applying scales specifically designed to measure QoL in our glaucoma clinic patient population to provide valuable insights that guide best practices for treatment and improved patient-centered care.

## Methods

The 25-item National Eye Institute Visual Function Questionnaire (NEI VFQ-25) and the Glaucoma Profile Instrument (GPI) surveys were administered to patients reporting for appointments at the Augusta University Eye Care Center from June to October 2023, prior to their ophthalmologist visit. Patients were consecutively recruited from this outpatient clinic during the study period and had the option to voluntarily decline participation. Subjects who were less than 18 years old, had cognitive impairment, or had altered mental status were excluded from the study. Non-English speakers and illiterate subjects were included in the study, although none of our subjects met these criteria. Participants who were blind or had severe visual impairment completed the survey with the assistance of a clinician who read the questions aloud.

Each patient’s medical charts were reviewed for demographic data, ophthalmologic diagnoses, glaucoma medications, visual acuity, and prior ophthalmologic surgeries. Based on their documented ICD-10 codes, the patients were classified as having glaucoma, being glaucoma suspects, or without glaucoma. The clinic followed the World Glaucoma Association guidelines for coding glaucoma stage, and the ICD-10 codes were chart validated at the time of the study. Treatment modality (medical vs. combined medical and surgical) was also recorded, along with types of surgical interventions when applicable. Surgical treatment included canaloplasty, trabeculotomy (OMNI Surgical System), transscleral cyclophotocoagulation (MicroPulse and G-probe), and glaucoma drainage implants (Ahmed FP7 valve and Baerveldt 350 sqmm implant). Cataract surgery was also included among the interventions, as the Ocular Hypertension Treatment Study showed it can significantly lower IOP, with an average 16.5% reduction from baseline [27]. Therefore, it is often considered an initial surgical procedure in glaucoma management. None of the included procedures during the time of this study involved the creation of an anterior filtering bleb.

The NEI VFQ-25 is a 25-question self-report survey designed to measure the progressive effect of visual impairment and chronic eye diseases on daily living and health-related quality of life. It is based on the original 51-question NEI VFQ, with similar reliability, validity, and sensitivity to glaucoma visual loss [28]. The 25 questions (seen in Online Resource 1) evaluate 11 “vision-related constructs” and a general health rating. The visual sub-scales generated from the 25 questions include global vision rating, difficulty with near vision activities, difficulty with distance vision activities, limitations in social functioning due to vision, role limitations due to vision, dependency on others due to vision, mental health symptoms due to vision, driving difficulties, limitations with peripheral and color vision, ocular pain, and a general health rating. The singular general health question serves as an indicator of health status and both morbidity and mortality in the future. This comprehensive coverage of QoL makes the VFQ-25 a great resource for our study [28]. The survey response scores are normalized according to the official scoring algorithm. Converted scores with this algorithm are reported as a percentage between 0 to 100% with higher percentage scores indicating better function [29].

The GPI is a six-question patient-reported outcome measure assessing glaucoma’s impact on QoL. The six questions (presented in Online Resource 2) address key aspects of daily functioning, such as visual performance, symptomatology, ability to perform activities of daily living, and other health-related effects [30]. The GPI serves to identify functional impairments caused by glaucoma in addition to the side effects of glaucoma treatment. Upon completion of the GPI, a Glaucoma Utility Index (GUI) can be derived, quantifying glaucoma’s influence on individual QoL [30]. This instrument is helpful for assessing glaucoma’s impact beyond the clinical use of IOP and visual field assessments. The GPI’s broad scope, including both the physical and psychosocial dimensions of glaucoma, makes it an invaluable tool for this study, offering a more comprehensive understanding of the disease’s overall burden on patients.

Associations between data extracted from chart review (race, sex, age, glaucoma status, glaucoma severity, treatment modality), the 12 VFQ-25 sub-scores, and the GPI score were analyzed. Patients were subdivided into three racial/ethnic categories (Black, White, and Other) and five age groups (50 years and below, 51–60 years, 61–70 years, 71–80 years, 81–90 years). All analyses were conducted using R version 4.4.1 [31]. The analysis of variance (ANOVA) was performed to determine each clinical variable’s association with the GPI score and VFQ-25 sub-scores. Subsequently, t-tests were conducted to compare each clinical variable to identify subgroups with significantly different effects on the scores.

## Results

A total of 180 patients with appointments at the Augusta University Eye Care Center from June to October 2023 consented to the study, and their data was evaluated. 9 subjects declined to participate. Of the 180 subjects, 103 were identified as patients with glaucoma: 29 subjects had suspected glaucoma, 14 had mild, 20 had moderate, and 40 had severe, based on ICD-10 codes and Humphrey visual field tests. 77 patients were identified as not having glaucoma. The average age across all subjects was 62.3 years. There were 104 female subjects, of which 53 have glaucoma. There were 76 male subjects, of which 50 have glaucoma. The primary racial composition of the subject pool was White/Caucasian and Black/African-American, with 60.6% of the African-American subjects having glaucoma and 48.5% of the Caucasian subjects having glaucoma. Within the subjects with mild, moderate, or severe glaucoma, 63.5% received only medical treatment, 33.8% received combined medical and surgical treatment, and 2.7% received no treatment (Table 1).

**Table 1:** Demographics of study population

		Glaucoma status				
	Total	No	Yes (n = 103)			
			Suspect	Mild	Moderate	Severe
	n = 180	n = 77	n = 29	n = 14	n = 20	n = 40
Age (mean)	62.3	62.3	61.0	64.9	65.7	60.9
*Gender*						
Female	104 (57.8%)	51 (66.2%)	17 (58.6%)	5 (35.7%)	14 (70.0%)	17 (42.5%)
Male	76 (42.2%)	26 (33.8%)	12 (41.4%)	9 (64.3%)	6 (30.0%)	23 (57.5%)
*Race/Ethnicity*						
White	68 (37.8%)	35 (45.5%)	9 (31.0%)	5 (35.7%)	6 (30.0%)	13 (32.5%)
Black	99 (55.0%)	39 (50.6%)	17 (58.6%)	7 (50.0%)	12 (60.0%)	24 (60.0%)
Other	13 (7.2%)	3 (3.9%)	3 (10.3%)	2 (14.3%)	2 (10.0%)	3 (7.5%)
*Treatment*						
None	99 (55.0%)	77 (100.0%)	20 (69.0%)	0 (0.0%)	1 (5.0%)	1 (2.5%)
Medical	55 (30.6%)	0 (0.0%)	8 (27.6%)	12 (85.7%)	12 (60.0%)	23 (57.5%)
Medical & Surgical	26 (14.4%)	0 (0.0%)	1 (3.4%)	2 (14.3%)	7 (35.0%)	16 (40.0%)

As shown in Table 2, there were no significant differences across the mean VFQ-25 composite scores amongst gender (*p* = 0.1952), glaucoma status (*p* = 0.5952), and treatment modality (*p* = 0.4021). In regard to gender, males (n = 76) scored a mean composite score of 71.36 ± 24.29, while females (n = 104) scored 75.57 ± 19.07. Subjects with no glaucoma (n = 77) scored 74.77 ± 19.25, while those with glaucoma (n = 103) scored 73.04 ± 23.08. Patients treated only with medicine (n = 55) had a mean composite score of 70.76 ± 24.73, while those also treated with surgery (n = 26) scored 65.90 ± 21.97. Comparison of NEI VFQ-25 mean composite scores showed significant differences in scores between race/ethnicity (*p* = 0.0489), severity (*p* = 0.0001), and age (*p* = 0.0459) subgroups. Black patients (n = 99) scored 74.41 ± 19.40, and White patients (n = 68) scored 70.51 ± 24.50, while patients of other race/ethnicity (n = 13) scored 86.19 ± 14.79. Glaucoma suspects (n = 29) scored the highest mean VFQ-25 composite score of 84.98 ± 17.36, while severe glaucoma subjects (n = 40) had the lowest score of 60.56 ± 25.51. Despite statistically significant differences, the effect sizes for VFQ-25 composite scores were negligible across severity categories when compared to individuals without glaucoma (Cohen’s d: –0.067 for suspects, –0.012 for mild, –0.019 for moderate, 0.084 for severe), indicating minimal clinical relevance. Within the age groups, those aged 71 and above (n = 51) scored highest at an approximate score of 80 ± 19. Those aged 61–70 years (n = 73) scored lowest at 69.13 ± 23.08. Although glaucoma severity was the only category with significant differences in mean GPI scores (*p* = 0.0092), the effect sizes remained small in all comparisons to the no-glaucoma group (Cohen’s d: 0.075 for suspects, –0.016 for mild, 0.049 for moderate, –0.041 for severe). All other categories (gender, race/ethnicity, glaucoma status, treatment modality, and age) did not reveal significant score differences amongst their subgroups.

**Table 2:** Comparison of composite NEI VFQ-25 and GPI scores across demographics

	Scoring method			
	VFQ-25 composite		GPI	
	Mean (95% CI)	*p-value*	Mean (95% CI)	*p-value*
Gender		0.1952		0.5151
Male (n = 76)	71.36 (23.42,119.30)		0.82 (0.53,1.12)	
Female (n = 104)	75.57 (37.95,113.20)		0.80 (0.50,1.11)	
Race/Ethnicity		0.0489		0.2401
Black (n = 99)	74.41 (36.14,112.69)		0.81 (0.51,1.11)	
White (n = 68)	70.51 (22.15,118.86)		0.80 (0.50,1.09)	
Other (n = 13)	86.19 (57.00,115.37)		0.88 (0.59,1.17)	
Glaucoma status		0.5952		0.5501
No (n = 77)	74.77 (36.78,112.76)		0.80 (0.50,1.10)	
Yes (n = 103)	73.04 (27.49,118.59)		0.82 (0.52,1.11)	
Severity		0.0001		0.0092
No glaucoma (n = 77)	74.60 (36.47,112.72)		0.80 (0.50,1.10)	
Glaucoma suspect (n = 29)	84.98 (50.73,119.23)		0.89 (0.66,1.12)	
Mild (n = 14)	76.50 (48.79,104.22)		0.78 (0.48,1.07)	
Moderate (n = 20)	77.63 (38.96,116.31)		0.86 (0.57,1.14)	
Severe (n = 40)	60.56 (10.23,110.90)		0.75 (0.45,1.04)	
Treatment Modality		0.4021		0.5195
Medical (n = 55)	70.76 (21.97,119.55)		0.80 (0.50,1.09)	
Medical & surgical (n = 26)	65.90 (22.55,109.25)		0.77 (0.48,1.06)	
Age		0.0459		0.2745
≤ 50 (n = 29)	70.47 (28.58,112.35)		0.79 (0.45,1.13)	
51–60 (n = 27)	78.19 (41.97,114.40)		0.85 (0.54,1.16)	
61–70 (n = 73)	69.13 (23.59,114.68)		0.79 (0.50,1.08)	
71–80 (n = 41)	80.06 (41.97,118.15)		0.84 (0.57,1.12)	
81–90 (n = 10)	80.19 (41.87,118.52)		0.77 (0.46,1.07)	

Table 3 evaluates the significance of differences between patients grouped by demographics (race, sex, age) and glaucoma factors (status, severity, and treatment modality) on the 12 sub-scores of the NEI VFQ-25 survey. Race demonstrates significant association with distance activity (*p* = 0.0152) and peripheral vision (*p* = 0.0194). Race reaches near significance for driving, near activity, dependency, and role difficulty. Sex only exhibited significant association with color vision (*p* = 0.0162). Age showed significant values in color vision (*p* = 0.0051), distance activity (*p* = 0.0370), dependency (*p* = 0.0482), mental health (*p* = 0.0269), and social function (*p* = 0.0216). Whether one has glaucoma (glaucoma status) does not significantly correlate with any NEI VFQ-25 subscore. This may have been due to the distribution of severity in glaucoma amongst the subjects, which showed statistically significant association with multiple NEI VFQ-25 segments: color vision (*p* = 0.0070), distance activity (*p* = 0.0001), driving (*p* = 0.0113), general vision (*p* = 0.0156), near activity (*p* = 0.0003), peripheral vision (*p* = 0.0000), dependency (*p* = 0.0004), mental health (*p* = 0.0007), role difficulty (*p* = 0.0299), and social function (*p* = 0.0006). Treatment modality, whether glaucoma was treated only medically or combined surgically and medically, was found to be significantly correlated with driving (*p* = 0.0378) and nearly significant for peripheral vision.

**Table 3:** Significance of demographics for each NEI VFQ-25 subscore

	vfq-25 subscore											
	Color vision	Distance activity	Driving	General health	General vision	Near Activity	Ocular pain	Peripheral vision	Dependency	Mental health	Role difficulty	Social function
Race	0.5614	0.0152	0.0774	0.6995	0.1977	0.0696	0.7689	0.0194	0.0889	0.1164	0.0698	0.1831
Sex	0.0162	0.9639	0.9430	0.9645	0.1995	0.2755	0.6112	0.8604	0.1579	0.3459	0.2118	0.1213
Glaucoma status	0.3218	0.7687	0.7025	0.6294	0.6092	0.9969	0.2019	0.5142	0.8967	0.9833	0.6977	0.6482
Severity	0.0070	0.0001	0.0113	0.1281	0.0156	0.0003	0.0724	0.0000	0.0004	0.0007	0.0299	0.0006
Treatment modality	0.3065	0.2207	0.0378	0.8922	0.7509	0.4703	0.6328	0.0836	0.5930	0.9011	0.4248	0.4772
Age	0.0051	0.0370	0.7707	0.7303	0.0901	0.1151	0.4395	0.1324	0.0482	0.0269	0.2586	0.0216

Within each demographic category, the differential impact of specific demographic components for each VFQ-25 subsection was evaluated, as seen in Table 4**.** Females had significantly higher color vision scores than males. There were no significant differences amongst patients of Black and White race, but patients of other race (Hispanic, Asian, Southeast Asian, Southwest Asian) had statistically significant higher scores than Black patients for distance activity, driving, near activity, peripheral vision, and mental health. All other sub-scores except for color vision, driving, general health, general vision, and ocular pain were significantly elevated in glaucoma suspects as compared to subjects with no glaucoma. Other than significant score decreases in general health, no other significant changes in scores were observed in mild glaucoma severity as compared to no glaucoma. Moderate glaucoma patients had no significant correlations with any sub-scores. Compared to subjects with no glaucoma, those with severe glaucoma had significant score decreases in multiple segments: color vision, distance activity, driving, general vision, near activity, ocular pain, peripheral vision, dependency, mental health, and social function. While 51–60 -year-old subjects had no significant score differences than those aged 50 or younger, those aged 61–70 had a significant score decline in color vision. Subjects aged 71–80 had positive score differentials in distance activity, dependency, and mental health. Finally, patients of age 81–90 years had a significant score increase compared to those age 50 or younger in general vision. No significant differences were seen in scores when comparing medical treatment to combined medical and surgical treatment.

**Table 4:** Differences amongst demographics for NEI VFQ-25 subscores

	VFQ-25 subscore											
	Color vision	Distance activity	Driving	General health	General vision	Near activity	Ocular pain	Peripheral vision	Dependency	Mental health	Role difficulty	Social function
Gender	Adj. Mean Diff (95% CI)	Adj. Mean Diff (95% CI)	Adj. Mean Diff (95% CI)	Adj. Mean Diff (95% CI)	Adj. Mean Diff (95% CI)	Adj. Mean Diff (95% CI)	Adj. Mean Diff (95% CI)	Adj. Mean Diff (95% CI)	Adj. Mean Diff (95% CI)	Adj. Mean Diff (95% CI)	Adj. Mean Diff (95% CI)	Adj. Mean Diff (95% CI)
Male (reference)												
Female	2.25 (0.27,4.23)*	0.04 (− 1.93,2.02)	− 0.07 (− 2.05,1.91)	− 0.04 (− 2.02,1.94)	1.26 (− 0.72,3.23)	1.07 (− 0.91,3.04)	0.50 (− 1.47,2.48)	0.17 (− 1.80,2.15)	1.39 (− 0.58,3.37)	0.94 (− 1.04,2.91)	1.24 (− 0.74,3.21)	1.50 (− 0.47,3.48)
Race/Ethnicity												
Black (reference)												
White	− 0.16 (− 2.14, 1.81)	− 1.85 (− 3.82,0.13)	0.35 (− 1.63,2.33)	− 0.37 (− 2.34,1.61)	− 1.21 (− 3.19,0.77)	− 0.82 (− 2.80,1.15)	0.50 (− 1.48,2.47)	− 1.02 (− 3.00,0.96)	− 1.56 (− 3.54,0.42)	− 0.72 (− 2.70,1.26)	− 1.63 (− 3.60,0.35)	− 1.24 (− 3.22,0.74)
Other	1.68 (− 0.37, 3.73)	2.43 (0.34,4.52)*	3.57 (1.49,5.65)*	0.72 (− 1.40,2.84)	1.63 (− 0.44,3.69)	2.32 (0.21,4.42)*	0.72 (− 1.39,2.83)	4.48 (2.46,6.51)*	1.25 (− 0.89,3.40)	2.13 (0.02,4.24)*	1.47 (− 0.68,3.63)	1.32 (− 0.79,3.42)
Glaucoma status												
No (reference)												
Yes	− 1.03 (− 3.00,0.95)	− 0.30 (− 2.27,1.67)	− 0.39 (− 2.37,1.59)	− 0.49 (− 2.46,1.49)	− 0.52 (− 2.50,1.45)	0.00 (− 1.97,1.98)	− 1.28 (− 3.26,0.69)	− 0.67 (− 2.64,1.31)	− 0.13 (− 2.11,1.84)	0.02 (− 1.95,2.00)	0.39 (− 1.59,2.37)	− 0.47 (− 2.44,1.51)
Severity												
No glaucoma (reference)												
Glaucoma SUSPECT	0.87 (− 1.15,2.88)	2.77 (0.77,4.76)*	1.79 (− 0.22,3.80)	1.00 (− 1.00,3.01)	1.34 (− 0.67,3.35)	2.74 (0.73,4.74)*	0.91 (− 1.09,2.91)	2.38 (0.38,4.38)*	3.26 (1.27,5.26)*	2.56 (0.55,4.56)*	2.39 (0.39,4.39)*	3.21 (1.22,5.21)*
Mild	1.99 (− 0.03,4.02)	1.19 (− 0.87,3.25)	0.36 (− 1.68,2.40)	− 2.17 (− 4.27,− 0.06)*	0.42 (− 1.68,2.51)	− 0.45 (− 2.54,1.64)	− 0.67 (− 2.77,1.43)	1.51 (− 0.56,3.59)	− 0.07 (− 2.15,2.02)	− 0.02 (− 2.11,2.07)	0.29 (− 1.79,2.37)	0.43 (− 1.64,2.50)
Moderate	− 0.57 (− 2.61,1.47)	0.54 (− 1.51,2.59)	0.72 (− 1.33,2.78)	0.16 (− 1.88,2.19)	0.56 (− 1.48,2.59)	1.15 (− 0.89,3.20)	− 0.92 (− 2.94,1.09)	0.08 (− 1.98,2.13)	0.82 (− 1.22,2.86)	1.10 (− 0.93,3.14)	1.11 (− 0.93,3.15)	0.14 (− 1.91,2.18)
Severe	− 2.40 (− 4.41,− 0.39)*	− 3.05 (− 5.05,− 1.05)*	− 2.22 (− 4.23,− 0.21)*	− 0.65 (− 2.64,1.35)	− 2.31 (− 4.30,− 0.31)*	− 2.54 (− 4.53,− 0.54)*	− 2.14 (− 4.14,− 0.15)*	− 3.36 (− 5.36,− 1.36)*	− 2.44 (− 4.44,− 0.44)*	− 2.46 (− 4.46,− 0.47)*	− 1.49 (− 3.49,0.50)	− 2.45 (− 4.45,− 0.45)*
Treatment modality												
Medical (reference)												
Medical & Surgical	− 0.98 (− 3.00,1.04)	− 1.29 (− 3.30,0.71)	− 1.96 (− 4.00,0.07)	0.14 (− 1.87,2.15)	0.32 (− 1.69,2.33)	− 0.79 (− 2.79,1.21)	0.45 (− 1.57,2.47)	− 1.84 (− 3.85,0.17)	− 0.57 (− 2.58,1.43)	0.14 (− 1.87,2.14)	− 0.79 (− 2.80,1.23)	− 0.79 (− 2.79,1.21)
Age												
≤ 50 (reference)												
51–60	0.02 (− 1.99,2.02)	1.42 (− 0.59,3.43)	0.96 (− 1.06,2.98)	0.12 (− 1.89,2.12)	2.09 (0.08,4.10)	0.51 (− 1.50,2.51)	1.05 (− 0.96,3.05)	1.05 (− 0.96,3.05)	1.40 (− 0.61,3.41)	1.35 (− 0.65,3.36)	1.55 (− 0.46,3.56)	0.86 (− 1.14,2.87)
61–70	− 2.61 (− 4.60,− 0.62)*	− 0.16 (− 2.17,1.84)	− 0.12 (− 2.14,1.90)	− 0.03 (− 2.04,1.99)	0.77 (− 1.23,2.78)	− 1.21 (− 3.21,0.80)	0.74 (− 1.26,2.74)	− 0.88 (− 2.89,1.12)	1.18 (− 0.83,3.19)	0.35 (− 1.66,2.36)	0.54 (− 1.47,2.55)	− 1.54 (− 3.54,0.46)
71–80	0.26 (− 1.74,2.26)	2.05 (0.04,4.05)*	0.04 (− 1.97,2.04)	1.01 (− 1.01,3.03)	1.65 (− 0.35,3.65)	0.77 (− 1.23,2.77)	1.97 (− 0.04,3.98)	0.58 (− 1.42,2.58)	3.00 (0.99,5.01)*	2.77 (0.76,4.77)*	1.82 (− 0.19,3.82)	0.88 (− 1.12,2.89)
81–90	1.20 (− 0.85,3.25)	1.23 (− 0.88,3.34)	0.60 (− 1.53,2.73)	− 0.11 (− 2.29,2.06)	2.54 (0.37,4.71)*	0.67 (− 1.48,2.81)	0.66 (− 1.47,2.79)	1.12 (− 1.03,3.28)	1.10 (− 1.00,3.20)	1.06 (− 1.05,3.17)	0.98 (− 1.12,3.07)	1.34 (− 0.70,3.38)

## Conclusion/discussion

In this clinical survey study, we found no significant difference in vision-related quality of life, as measured by the NEI VFQ-25 survey, between individuals treated medically versus those treated with combined medical and surgical management for glaucoma. Most subjects opted for only medical treatment over surgery, regardless of disease severity, with an average use of two or more medication classes. Given treatment modality does not definitively impact QoL, this may offer clinical validity to why most patients in our study did not undergo surgical treatment.

Notable differences in QoL were observed when glaucoma severity was considered, emphasizing the disease’s progressive nature and supporting the NEI VFQ-25 as a valid tool for tracking QoL across disease severities. However, the effect size was limited. This may be due to the need for a larger study population with sufficient power to detect differences across more specific subgroups. Future studies should consider stratifying patients by treatment intensity, such as the number of topical medications used, and by surgical type, including trabeculectomy, tube shunts, and various MIGS procedures. Those with suspected or severe glaucoma displayed measurable QoL differences compared to those without glaucoma. Differences were also observed across age, race/ethnicity, and gender, suggesting that disease severity and unmeasured factors may play larger roles in QoL outcomes. Our findings reinforce that glaucoma care must be holistic, addressing comorbidities and broader health challenges [18].

While general health scores from the NEI VFQ-25 predict future health and mortality, our study, like previous studies, found no meaningful relationship between general health scores and glaucoma severity, treatment type, or demographics [32]. Similar to our findings, previous studies have found that subcategories such as driving, mental health, peripheral vision, and social functioning aligned more closely with disease impact [28, 32]. Color vision was the only category with a sex-based difference: females had significantly higher ratings, consistent with studies showing better color perception among women [33]. Despite more severe disease often being observed in Black patients, we found no significant racial differences in QoL [34]. No significant QoL differences were found between treatment modalities, though poorer scores reported in the surgical group may reflect greater disease burden [17]. As expected, patients with severe glaucoma, or once vision loss became particularly noticeable, had significantly decreased QoL scores [35, 36].

Since both surgical and medical treatments had minimal impact on QoL, this highlights the need to revise strategies for counseling patients, particularly those with uncontrolled intraocular pressure. Counseling should also recognize that surgery may help prevent progression but does not appear to improve QoL meaningfully. Innovation in glaucoma treatments, such as microinvasive surgery, novel drug-delivery implants, or vision-assistive technology, may offer better solutions to enhance QoL, especially for severe cases [37]. It is also possible that poorer QoL in surgically treated patients reflects more advanced disease rather than treatment effects, a form of reverse causality [38, 39]. Additionally, patients using multiple IOP-lowering medications, such as those in our study, may have QoL outcomes worse than those on monotherapy [40, 41]. Furthermore, unmeasured factors, such as patient expectations, treatment satisfaction, adherence, or perceived disease control, may influence reported QoL and can influence the results as confounding factures. This may explain the lack of significant differences between groups. Finally, while surgery may not improve QoL, it may serve to preserve existing QoL and prevent further decline [42]. In clinical practice, incorporating QoL assessments into routine glaucoma management can help capture patient-reported impacts beyond visual field measures. Clinicians should interpret these findings alongside disease severity to guide individualized counseling and treatment decisions. Emphasizing patient education, adherence, and mental well-being may help preserve QoL even in advanced disease stages.

There are some limitations to our study results. The NEI VFQ-25 and GPI surveys may not fully capture all aspects of glaucoma’s effects on QoL of our Augusta, Georgia population, particularly given local socioeconomic and healthcare access disparities. These challenges may influence how patients perceive and report their QoL [43, 44]. As a result, our findings may not be fully generalizable to other glaucoma patient populations or care settings with different demographic, healthcare, socioeconomic, or geographical characteristics. Self-reporting can further introduce bias due to patient recall issues or subjective perceptions [45]. Selection bias may have influenced the results because patients who underwent surgical management may have more severe or treatment-resistant glaucoma, greater visual field loss, lower baseline QoL, lower medication adherence, or other unmeasured baseline differences compared with those treated only medically. These factors could obscure potential benefits of surgery in cross-sectional comparisons. However, with only 5% of subjects declining participation and no reported issues related to literacy or language, the risk of voluntary response bias appears minimal. In addition, a larger study population may be required to assess whether different types of surgery have varying impacts on QoL. Finally, the cross-sectional study design limits the ability to draw causal inferences from these findings. The directionality of the observed associations cannot be determined, and reverse causality, such as worse QoL leading to surgical intervention, remains possible. Future longitudinal or experimental studies are needed to better establish causal relationships.

In conclusion, patients with varying severities of glaucoma experienced differential impacts to aspects of their vision-related QoL. Whether through medicine or surgery, the different treatment modality showed negligible effects on QoL. Future studies are necessary to determine why standard glaucoma treatment regimens do not contribute to increased QoL, and innovative treatment discovery should be encouraged to help significantly improve QoL for glaucoma patients.

## Supplementary Information

Below is the link to the electronic supplementary material.

Supplementary file1 (PDF 144 kb)

## Data Availability

The data that support the findings of this study are not openly available due to reasons of sensitivity and are available from the corresponding author upon reasonable request.
