# Supplementary material for: The impact of treatment modality on quality of life in glaucoma patients: findings from a clinical survey study in Augusta, GA
Source: Int Ophthalmol. 2025 Dec 29;46(1):55. doi: 10.1007/s10792-025-03895-7 (PMC12748089; doi:10.1007/s10792-025-03895-7)
Supplement: Supplementary file 1 — Supplementary file1 (PDF 144 kb) [file 10792_2025_3895_MOESM1_ESM.pdf]

Supplementary Information

Online Resource 1. Questions Asked by the NEI VFQ-25 Questionnaire

NEI VFQ-25 Questions
1. In general, would you say your overall health is:
2. At the present time, would you say your eyesight using both eyes (with glasses or contact lenses, if you wear them) is excellent, good, fair, poor, or very poor or are you completely blind?
3. How much of the time do you worry about your eyesight?
4. How much pain or discomfort have you had in and around your eyes (for example, burning, itching, or aching)? Would you say it is:
5. How much difficulty do you have reading ordinary print in newspapers? Would you say you have:
6. How much difficulty do you have doing work or hobbies that require you to see well up close, such as cooking, sewing, fixing things around the house, or using hand tools? Would you say:
7. Because of your eyesight, how much difficulty do you have finding something on a crowded shelf?
8. How much difficulty do you have reading street signs or the names of stores?
9. Because of your eyesight, how much difficulty do you have going down steps, stairs, or curbs in dim light or at night?
10. Because of your eyesight, how much difficulty do you have noticing objects off to the side while you are walking along?
11. Because of your eyesight, how much difficulty do you have seeing how people react to things you say?
12. Because of your eyesight, how much difficulty do you have picking out and matching your own clothes?
13. Because of your eyesight, how much difficulty do you have visiting with people in their homes, at parties, or in restaurants ?
14. Because of your eyesight, how much difficulty do you have going out to see movies, plays, or sports events?
15. Are you currently driving, at least once in a while?
15a. IF NO: Have you never driven a car or have you given up driving?
15b. IF YOU GAVE UP DRIVING: Was that mainly because of your eyesight, mainly for some other reason, or because of both your eyesight and other reasons?
15c. IF CURRENTLY DRIVING: How much difficulty do you have driving during the daytime in familiar places? Would you say you have:
16. How much difficulty do you have driving at night? Would you say you have:
16A. How much difficulty do you have driving in difficult conditions, such as in bad weather, during rush hour, on the freeway, or in city traffic? Would you say you have:
17. Do you accomplish less than you would like because of your vision?
18. Are you limited in how long you can work or do other activities because of your vision?
19. How much does pain or discomfort in or around your eyes, for example, burning, itching, or aching, keep you from doing what you'd like to be doing?
20. I stay home most of the time because of my eyesight.
21. I feel frustrated a lot of the time because of my eyesight
22. I have much less control over what I do, because of my eyesight.
23. Because of my eyesight, I have to rely too much on what other people tell me
24. I need a lot of help from others because of my eyesight.
25. I worry about doing things that will embarrass myself or others because of my eyesight

Online Resource 2. Questions Asked by the GPI Questionnaire

GPI Questions
Mark for each question which best describes any difficulties you have had in the last month with your eyes or vision with answers ranging from none, some, quite a lot, and severe:
1. Central and near vision (ie. difficulties with reading, watching TV and computer use)
2. Lighting and glare (ie. difficulties with adjusting from light to dark, bright lights, difficulties seeing in dim light)
3. Mobility (ie. difficulties with crossing roads, driving, negotiating steps)
4. Activities of daily living (ie. difficulties with household or DIY tasks, pouring liquids into containers, shaving, etc)
5. Eye discomfort (ie. difficulties with gritty, sore, tired eyes)
6. Other effects (ie. fatigue, shortness of breath, dry mouth, bitter taste)

Article Title: The Impact of Treatment Modality on Quality of Life in Glaucoma Patients: Findings from a Clinical Survey Study in Augusta, GA

Journal Name: *International Ophthalmology*
Author Names: Neel Edupuganti, Haley Chishom, Tommy Bui, Danny Alevy, Tae Jin Lee, PhD, Mathilde Franklin, MD, Marc Töteberg-Harms, MD

Corresponding Author:
Marc Töteberg-Harms, MD
University of Iowa, Carver College of Medicine
Department of Ophthalmology & Visual Sciences
200 Hawkins Dr, Iowa City, IA 52242
Marc-Toeteberg-Harms@uiowa.edu
